# Partial nephrectomy without prior arterial embolization in a case of giant renal angiomyolipoma

**DOI:** 10.1016/j.ijscr.2024.110182

**Published:** 2024-08-18

**Authors:** Yamen Al Ahmad, Khaled T. Dardeer, Akram Wafiq Abo Daken

**Affiliations:** a Urology Department, Faculty of medicine, Damascus University, Syria; b Faculty of medicine, Cairo university, Cairo, Egypt; c Head of emergency and inpatient department, Faculty of medicine, Damascus University, Syria

**Keywords:** Partial nephrectomy, Angiomyolipoma, Oncology

## Abstract

**Introduction and importance:**

This is a case of giant renal angiomyolipoma treated with partial nephrectomy without pre-embolization of the supplying arteries.

**Case presentation:**

The patient presented for regular checkup, a mass in the left flank was found on examination. No other symptoms were present. No history of medical or surgical importance was found.

**Clinical discussion:**

CT scan revealed a heterogenous mass in the left kidney at the upper pole. The treatment options were discussed with the patient and he opted for complete removal of the mass. Partial nephrectomy was done without pre embolization of the arteries. The procedure went without complications, the blood loss was manageable during the operation (200 cc). The patient was admitted for 48 h post operation and was discharged shortly after. The excised mass was sent for histopathological examination, the report was received, the features of the mass was consistent with angiomyolipoma without other types of malignant masses present.

**Conclusion:**

The takeaway is that partial nephrectomy provides a convenient option for treatment of giant renal angiomyolipomas. Preservation of the renal function, reduction of the recurrence risk or re embolization of the supplying arteries makes it a viable alternative for other treatment modalities.

## Highlights

• Renal angiomyolipomas can grow to large sizes and be completely symptomless until a hemorrhage event occurs.
• Partial nephrectomy provides a viable and safe alternative to embolization and complete nephrectomy.
• Prior embolization of feeding vessels is not required for a successful, complication-free operation.

## 1 Introduction

Angiomyolipomas are benign neoplasms consisting mainly of 3 components: blood vessels, fatty adipose tissue, and spindle smooth muscle cells. They are most commonly present in the kidneys, less commonly in the liver and other organs [1]. Renal angiomyolipoma belong to a family of rare tumors named perivascular epithelioid cell neoplasms (PEComas), characterized by the presence of epithelioid cells around the blood vessel. Angiomyolipomas are usually highly vascular, with a great risk of bleeding during operation [2].

Renal angiomyolipomas are the most prevalent benign renal tumors [3]. However, they are rare tumors overall, accounting for roughly 0.3 % to 3 % of all renal neoplasms. The incidence in females is higher compared to males (2:1 ratio), presumably due to estrogenic effects. The mean age of presentation is around 43 years, and usually occur as a sporadic incidence (80 %) [2].

Usually, renal angiomyolipoma present with no symptoms; the discovery of such tumors is often incidental during routine checkup or through radiology indicated for other reasons [4]. Common symptoms include flank pain and presence of “heaviness” or mass in the abdomen. On the other hand, severe presentations include shock and retroperitoneal hemorrhage due to spontaneous rupture of the tumor. The main clinical concern is the possible severe bleeding, especially in large-sized tumors.

In addition, several studies hinted at a link between sex hormones and angiomyolipomas. Boorijan and colleagues found estrogen, progesterone and androgen receptors on specimens of angiomiolypomas [5]. In another study, the authors found increased incidence of AMLs in women in addition to reports of AML growth during pregnancy and in patients taking contraceptive hormones [6].

Diagnostic radiology is the main cornerstone for identifying angiomyolipomas. Several radiological modalities can aid in the diagnosis of such tumors. Ultrasound shows hyperechoic lesions with posterior shadow, a characteristic appearance of fat-rich angiomyolipomas. However, a pitfall with US is the common confusion with renal cell carcinomas (RCC); as around a third of RCCs share a common appearance on ultrasound [4,7].

CT and MRI are similar in accuracy for detecting angiomyolipomas, with MRI having a little edge over CT for detecting fat-poor lesions [8]. Percutaneous renal biopsy is used mainly in differentiating RCCs from fat-poor or fat-invisible lesions when both CT and MRI are inconclusive [2,9].

Angiomyolipomas are mostly asymptomatic, the usual management for asymptomatic cases is follow up. Currently, the main indications for active treatment are as follows: >6 cm in size, symptomatic, women in childbearing age, >5 cm intralesional aneurysm, >2.5 mm enlargement rate per year, and other patient-related factors such as poor compliance, poor access to emergency care and high-risk occupations [10].

Treatment modalities include medical and invasive surgical procedures. mTOR inhibitors can be used to treat hereditary angiomyolipomas >3 cm of size, and help promote the regression of the lesion [11]. Thermal ablation and cryotherapy could be used in small renal masses <3 cm. However, radiofrequency ablation is preferred for treatment of small-sized lesions; the technique is minimally invasive with adequate effectiveness [12].

Surgical procedures include selective renal artery embolization, total nephrectomy and partial nephrectomy. Selective embolization is currently considered the first line treatment, especially if risk of acute hemorrhage is present [13]. Total or partial nephrectomy are the only treatment options that completely remove the renal mass, and prevent possible recurrence or complications. Partial nephrectomy could be done through laparoscopic or open approach. Nephrectomy is indicated if the suspicion of malignancy is present, the mass is large in size, or the other treatment options are not available [10]. This case report is compliant with SCARE guidelines [14].

## 2 Case report

We report a case of 38 years old male, single, with no prior surgical or medical history. The patient presented for routine checkup at Faculty of medicine, Damascus university. Physical examination revealed no abnormal findings, except for a mass in the left flank. The patient was vitally stable, and reported no associated symptoms such as flank pain, abdominal heaviness or mass. In addition, no symptoms indicative of urinary tract affection were present.

**Fig. 1:**
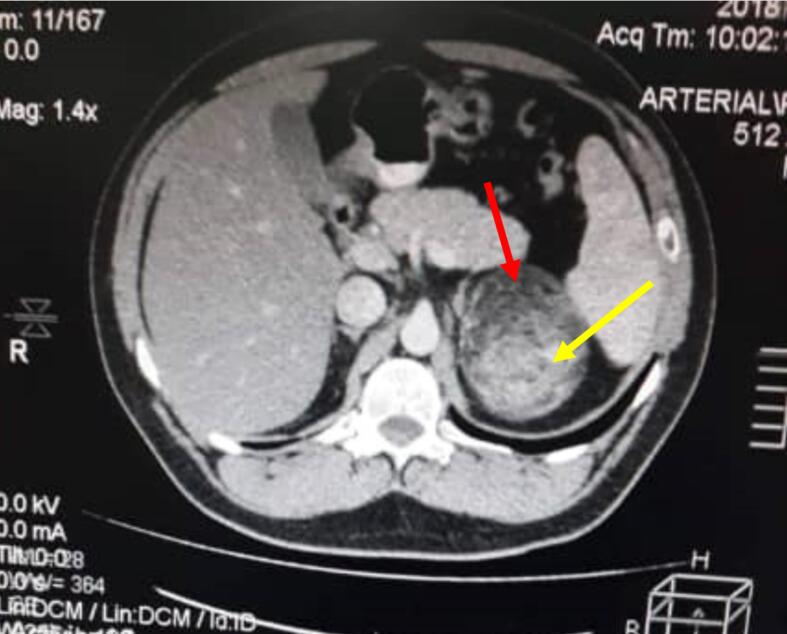
Contrast enhanced axial abdominal CT scan showing the unilateral AML mass. The mass is heterogenous due to mixed composition. Red arrow shows the fat component which appears hypodense (darker) on the CT scan, while the yellow arrow shows the hyperdense vascular and muscle tissue (brighter) on CT scan due to uptake of contrast material. The mass is well-circumscribed, the perimeter can be traced without discontinuations. (For interpretation of the references to colour in this figure legend, the reader is referred to the web version of this article.)

Further investigations in the form of labs and radiology were requested. Labs in the form of complete blood count, INR, kidney and liver functions revealed no abnormalities, the pre and post operative lab data are present in Table 1. CT scan revealed a mass 8*7 cm at the upper pole of the left kidney. Preliminary diagnosis of renal angiomyolipoma was made based on the characteristic appearance on the CT scan (Fig. 1).

**Table 1:** Pre and post operative lab data of the patient.

Parameter	Pre-operative	Immediate post operative	Ref range
RBCs (million/μL)	5.16	4.89	4.5–5.5
Hb (g/dL)	15.4	14.2	13–17
Hct (%)	44.8	42.2	40–50
MCV (fL)	86.8	91.9	76–96
MCH (pg)	29.8	30.9	27–32
MCHC (g/dL)	34.4	33.6	31.5–34.5
PLT (Thousand/μL)	287	307	150–400
TLC (thousand/μL)	9.5	10.8	4–10
Neutrophils (%)	62.7	51.9	–
Lymphocytes (%)	27.9	37	–
Eosinophils (%)	0.3	1.8	–
Basophils (%)	0.3	0.4	–
Monocyte (%)	8.8	8.9	–
PT (seconds)	15.5	13.1	11–16
INR	1.17	1.02	0.9–1.4
AST (U/L)	17	25	up to 38
Creatinine (mg/dL)	1.17	0.95	0.7–1.3
Na (mEq/L)	143	141	136–145
K (mEq/L)	3.6	4.1	3.5–5.1

Initial treatment options included selective arterial embolization, partial or total nephrectomy. Nephrectomy was chosen based on the size of the tumor, and a decision to spare the kidney and perform a partial nephrectomy was made. No pre operative embolization was done, the amount of blood loss was manageable during the operation (∼200 cc). Complete excision of the mass was performed, and the recovery process went uneventful. The patient was discharged following a period of 48 h in the hospital**.**

**Fig. 2:**
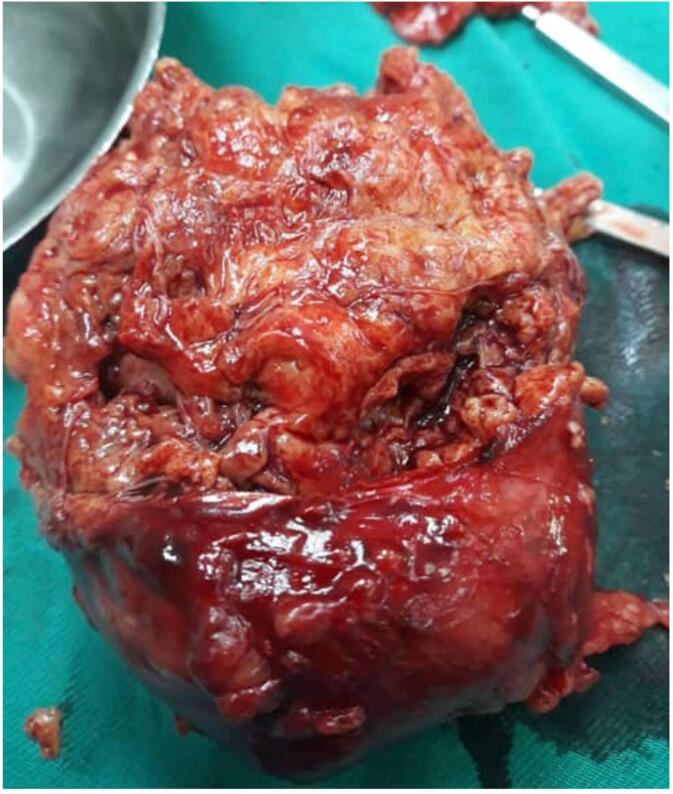
Picture of the excised renal AML mass. The cut section shows lobulated yellowish tissue consisting primarily of fat lobules, a characteristic of renal AMLs. The tumor measures 9 × 8 × 6 cm.

The specimen was sent for complete histopathological examination. The pathology report revealed a tumor ∼10 cm in the largest dimension, with yellowish lobulated cut-section. Multiple fragments of soft tissues weighing 330 g was discovered, the largest was a partially capsulated mass measuring 9 ∗ 8 ∗ 6 cm (Fig. 2).

Annual follow up for the patient for 4 years revealed no early or late post operative complications and no signs of tumor recurrence. The patient was considered disease free based on clinical follow up.

## 3 Discussion

In this report, we present a case of sporadic giant renal angiomyolipoma. Renal angiomyolipomas may present as sporadic, or associated with other conditions such as tuberous sclerosis complex (TSC) or pulmonary lymphangioleiomyomatosis [2].

However, no history of any symptoms suggesting the involvement of these 2 conditions were present in this patient. The management for angiomyolipomas include non-invasive medical treatment or invasive surgical operations. Medical treatment revolves mainly around mTOR inhibitors, these are primarily used in hereditary renal angiomyolipomas associated with TSC or pulmonary lymphangioleiomyomatosis, which make them out of the question for this patient [15].

On the other hand, several options are available for surgical procedures, the least invasive of which is selective trans arterial embolization (TAE). TAE is widely regarded as the first line treatment of renal AMLs. Minimal invasiveness, high success rate, and preservation of the renal function makes it a favorable procedure for clinicians [16]. However, it is not without downsides, some authors expressed concern for the long-term efficacy of the procedure [17]. Moreover, high rates of side effects were observed in some studies, and up to %30 of patients will need second treatment due to revascularization of the tumor [10,17].

These factors have been discussed with the patient, and he opted for surgical excision for complete removal of the mass and to avoid the possible need for retreatment. Surgical excision entails complete and partial nephrectomy. Complete nephrectomy is preserved for malignant masses and patients with severe life-threatening hemorrhage that would not be stopped with embolization [10].

Partial nephrectomy preserves the renal functions, significantly decreases recurrence, and has an acceptable rate of complications [10]. It is worth noting that laparoscopic or open approach could be used in partial nephrectomy, in our case, we chose the open approach due to the large size of the tumor. In this patient, the procedure went without any significant complication, and the volume of blood loss was manageable (200 cc). Blood loss was estimated by several procedures: Weighting any used surgical sponges and gauzes and measuring the amount of blood measured in the suction device. Although we did not do pre embolization of the tumor, some authors suggest that pre operative embolization helps to reduce the tumor size, and decrease the risk of intraoperative bleeding [9].

Several studies found concurrent presence of AML and a malignant mass in the same kidney. We sent the excised mass for complete histopathological examination to exclude the presence of malignant foci, and the report came back negative for any malignancy [18].

Some authors found that recurrence was found to happen following partial nephrectomy, albeit rarely. While no definitive guidelines for the follow up of AMLs are established, studies suggest that annual radiological for solitary lesions are recommended [7]. The patient was followed up clinically and radiologically for any signs of recurrence or possible missed metastatic foci; the follow up was negative for a period of 4 years.

## 4 Conclusion

In this case of large renal angiomyolipoma, partial nephrectomy was a successful intervention. The procedure is effective for sporadic renal AMLs, and constitutes a viable alternative to trans arterial embolization.

## Informed consent to participate and publish

The participant has consented to the writing and submission of the case report to the journal.

## Ethical approval

To the authors knowledge, IRB approval is not required in a case report with de-identified information.

## Funding

No funding was received for conducting this study.

## Author contribution

Yamen Al Ahmad: Conceptualization of the study idea, performance of the clinical procedure, supervision and observation of the writing process.

Khaled T. Dardeer: Writing and editing of the manuscript, final review and submission process.

Akram Wafiq Abo Daken: Conceptualization of the study idea, performance of the clinical procedure, supervision and observation of the writing process.

## Guarantor

Dr. Yamen Al Ahmad.

## Research registration number

1. Name of the registry:researchregistery.com.
2. Unique identifying number or registration ID: researchregistry10238.
3. Hyperlink to your specific registration (must be publicly accessible and will be checked): https://researchregistry.knack.com/research-registry#home/registrationdetails/662f1e47ba66a900298222a3/.

## Conflict of interest statement

The authors report no conflicts of interest.

## Data Availability

Data for this study is available upon reasonable request with the corresponding author.

## Appendix A Supplementary data

Supplementary material Image 1
