# Supplementary material for: Partial nephrectomy without prior arterial embolization in a case of giant renal angiomyolipoma
Source: Int J Surg Case Rep. 2024 Aug 18;123:110182. doi: 10.1016/j.ijscr.2024.110182 (PMC11400986; doi:10.1016/j.ijscr.2024.110182)
Supplement: Supplementary file 1 — Supplementary material [file mmc1.docx]

**Preoperative Management**

- - **Medical History and Physical Examination**: complete medical history was taken and clinical examination was done prior to the
  - **Laboratory Tests**: CBC, renal function tests, coagulation profile, and electrolyte levels were conducted as detailed in Table.1.
  - **Imaging Studies**: Contrast enhanced CT scan was performed to visualize the tumor mass.
  - **Consultations** were not required in this patient as no significant comorbidity was found during the examination.
  - **Antibiotics:** Cefazolin was administered 1g IV 60 mins before the procedure

**Intraoperative Management**

- - **General Anesthesia** was performed as it is the standard practice for partial nephrectomy (Zhao et al., 2016).
  - **Urinary Catheter**: A Foley catheter was following induction of general anesthesia to monitor urine output.

**Postoperative Management**

- - **ICU admission was not required in this patient as no intraoperative complications were present**
  - **Patient was low risk for VTE; no LMWH was given. Early mobilization was the method of choice for prophylaxis against thromboembolisms**

**Postoperative Mobilization**:

- - **Timing**: Mobilization was done 12hr post operative to avoid risks of VTE.
  - **Physiotherapy** was not required in this patient as no operative or post operative complications were observed.

**Urine Laboratory Check-up**:

- - **Urine analysis** was conducted 24 hrs after the operation when the urine output of the patient was clear in the following table

**Table.S1 Laboratory urine analysis data of the patient**

| Physical examination | |
| --- | --- |
| Color | Pale yellow |
| Appearance | Clear |
| Chemical examination | |
| Specific gravity | 1024 |
| pH | 5.6 |
| Protein | null |
| Glucose | null |
| Ketones | null |
| Bilirubin | null |
| Microscopic examination | |
| RBCs | 6 - 8 |
| WBCs | 4 - 6 |
| Epithelial cells | + |
| Casts | null |
| Calcium Oxalate | + |
| Uric acid | + |
| Bacteria | null |
| Yeast | null |
| Ova | null |

- - **Renal Function Tests**: KFTs were repeated post operatively and found to be within normal values

**Hospital stay:**

- - Patient was admitted to the surgical ward as ICU admission was deemed unnecessary
  - Patient was monitored for 48 hrs and was discharged when the general condition was well enough and no complications were observed.

**Surgical Technique for Open Partial Nephrectomy (adapted from (Zhao et al., 2016)**:

(A) After achieving vascular control, an incision is made in the kidney capsule, leaving a margin of about 5 mm.

(B) The renal artery is clamped using a bulldog clamp, and the tumor is excised. Any visible vessels are clipped or tied off.

(C) The blood vessels and the collecting system are sutured closed with a continuous suture, and the bulldog clamp is then released from the renal artery.

(D) Renorrhaphy involves bringing the cortical edges together with interrupted sutures and applying clips as needed.

(E) These interrupted sutures are secured over a bolster, such as a piece of absorbable cellulose or a flap of perinephric fat.

- - Zhao, P. T., Richstone, L., & Kavoussi, L. R. (2016). Laparoscopic partial nephrectomy. *International Journal of Surgery*, *36*, 548-553. <https://doi.org/https://doi.org/10.1016/j.ijsu.2016.04.028>
